# Association Between Intraoperative Hypothermia and Preoperative Body Composition in Patients Undergoing General Anesthesia for Gastrointestinal Surgery

**DOI:** 10.7759/cureus.88258

**Published:** 2025-07-18

**Authors:** Shuta Sugiyama, Yuta Mitobe, Moe Sakata, Kenta Akitsu

**Affiliations:** 1 Department of Nursing, International University of Health and Welfare (IUHW) Atami Hospital, Atami, JPN; 2 Perianesthesia Nursing, International University of Health and Welfare, Tokyo, JPN; 3 Department of Radiology, International University of Health and Welfare (IUHW) Atami Hospital, Atami, JPN

**Keywords:** ct images, gastrointestinal surgery, perioperative hypothermia, preoperative body composition, subcutaneous adipose tissue index

## Abstract

Introduction

General anesthesia induces a redistribution of deep body temperature drop, and a decrease in body temperature during surgery is associated with increased blood loss and transfusion requirements, increased incidence of wound infections, and prolonged hospital stays. Furthermore, low body mass index (BMI) is associated with a pronounced decrease in body temperature. However, subcutaneous fat mass, visceral fat mass, and skeletal muscle mass have not been precisely measured. More precise quantification of body composition can be achieved using computed tomography (CT) imaging, which allows the measurement of total fat, subcutaneous fat, visceral fat, and subcutaneous fat areas. Therefore, this study aimed to investigate whether differences in body composition obtained from CT images affect intraoperative temperature variations.

Methods

This was a single-center, retrospective observational study. The participants were Japanese patients who underwent elective gastrointestinal surgery under general anesthesia between April 1, 2021, and March 31, 2022. Hypothermia was defined as a core temperature of less than 36.0℃. Body temperature during surgery was measured by pharyngeal or bladder temperature, and patients who had a temperature of < 36.0℃ for at least one minute during anesthesia were classified into the hypothermia group, and all other patients were assigned to the control group. We investigated the relationship between fat mass, visceral fat mass, and skeletal muscle mass measured from preoperative CT images, along with preoperative blood sampling data and intraoperative body temperature. To standardize the estimated area, the index was calculated by dividing the heights of the two surpluses. The subcutaneous adipose tissue index (SATI) is the cross-sectional area of subcutaneous fat (cm^2^) divided by height^2^ (m^2^), and the visceral adipose tissue index (VATI) is the visceral adipose cross-sectional area (cm^2^) divided by height^2^ (m^2^). Statistical analysis was performed using the Mann-Whitney U test to compare the hypothermia and control groups. Logistic regression analysis was performed to identify the factors showing significant differences. Furthermore, receiver operating characteristic (ROC) analysis was performed, and the cutoff values were calculated. The significance level was set at p < 0.05.

Results

The analysis included 35 patients each in the hypothermia and control groups. SATI (cm²/ⅿ²) was significantly lower in the hypothermia group (median 27.91), compared to the control group (median 37.83) (p=0.033). VATI (cm²/ⅿ²) did not significantly differ between the hypothermia (median 32.62) and control groups (median 32.53) (p=0.828). Similarly, SMI (cm²/ⅿ²) exhibited no significant differences between the hypothermia (median 31.93) and control groups (median 34.65) (p=0.19). Univariate and multivariate logistic regression analyses were performed to identify the risk factors associated with the development of hypothermia. SATI was a significant factor in both univariate (OR: 5.45, 95%CI: 1.960-15.20, p=0.01) and multivariate analyses (OR: 5.520, 95%CI: 0.199-15.50, p> 0.001).

Conclusions

A decreased SATI was associated with intraoperative hypothermia in patients undergoing gastrointestinal surgery. Preoperative CT-based measurements of SATI may serve as a useful tool for predicting the risk of intraoperative hypothermia.

## Introduction

General anesthesia induces a redistribution of deep body temperature drop [1], and a decrease in body temperature during surgery is associated with increased blood loss and transfusion requirements, increased incidence of wound infections, and prolonged hospital stays [2-4], leading to adverse patient outcomes. Additionally, maintaining normothermia has been shown to be cost-effective and associated with improved patient outcomes [5]. Previously reported predictors of hypothermia include small body weight vs. tolerated surface area, age, height, weight, systolic blood pressure, heart rate, and use of intraoperative hypertensive drugs [6-8]. Furthermore, low body mass index (BMI) is associated with a pronounced decrease in body temperature [9].

However, subcutaneous fat mass, visceral fat mass, and skeletal muscle mass have not been precisely measured. More precise quantification of body composition can be achieved using computed tomography (CT) imaging [10], which allows the measurement of total fat, subcutaneous fat, visceral fat, and subcutaneous fat areas [11]. Previous studies indicate that information obtained from body composition is related to the decrease in body temperature during surgery, particularly due to the high insulating effect of the patient's adipose tissue. A small decrease in body temperature has been observed in patients with higher adipose tissue, and visceral fat is associated with a decrease in body temperature [12,13]. We did not find any reports on the association between skeletal muscle mass and intraoperative hypothermia.

Therefore, this study aimed to investigate whether differences in body composition obtained from CT images affect intraoperative temperature variations. The findings of this study suggest the possibility of predicting intraoperative temperature variations before and during surgery, which may lead to safer intraoperative patient management.

## Materials and methods

This was a single-center, retrospective observational study conducted at the International University of Health and Welfare (IUHW) Atami Hospital, Atami, Japan. The study was approved by the Ethical Review Committee of the International University of Health and Welfare, Atami Hospital (approval number: 22-A-215). This study was conducted in accordance with the principles of the Declaration of Helsinki. An “Information Disclosure Statement” clearly outlining the ethical considerations and allowing patients to withdraw from participation at any time was posted on the website of Atami Hospital, International University of Health and Welfare, Atami, Japan.

Eligibility criteria

The participants were Japanese patients who underwent elective gastrointestinal surgery under general anesthesia between April 1, 2021, and March 31, 2022. The surgical procedures included both laparoscopic and open approaches. Among these, patients aged ≥ 18 years who underwent surgery for ≥ two hours and for whom preoperative CT images, including the L3 region, were obtained for body composition measurement [14], were included. Patients were excluded if they had not undergone CT imaging, if CT-based body composition analysis was difficult, if they underwent colostomy, had an American Society of Anesthesiologists Physical Status (ASA-PS) score ≥ 3, had a Clavlen-Dindo classification of IIIa or higher, or if the surgical procedure involved thoracic cavity manipulation [15].

Data collection and study procedure

**Figure 1:**
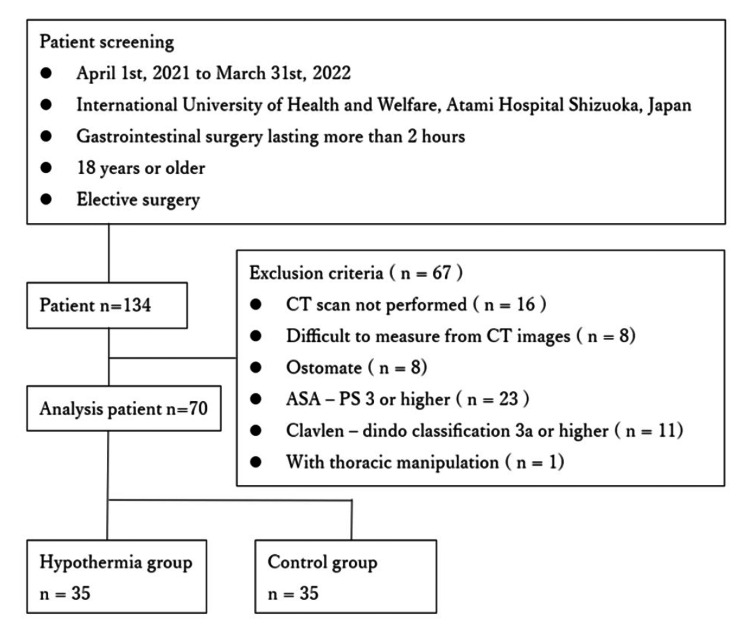
Flowchart of analysis study population CT: computed tomography, ASA-PS: American Society of Anesthesiologists Physical Status

General anesthesia was administered by an anesthesiologist certified by the Japanese Society of Anesthesiologists. Body temperature during surgery was obtained from anesthesia records, measured by either pharyngeal or bladder temperature. Hypothermia was defined as a core temperature of less than 36.0℃ [16]. Patients whose body temperature remained below 36.0°C for at least one minute during anesthesia were classified into the hypothermia group, and all other patients were assigned to the control group (Figure 1).

All patient grouping was performed retrospectively by the principal investigator after the completion of data collection. Patients were categorized based on predefined criteria described in this section. The grouping process was conducted before the statistical analysis and without knowledge of the study outcomes to minimize potential bias.

CT imaging-based measurements

**Figure 2:**
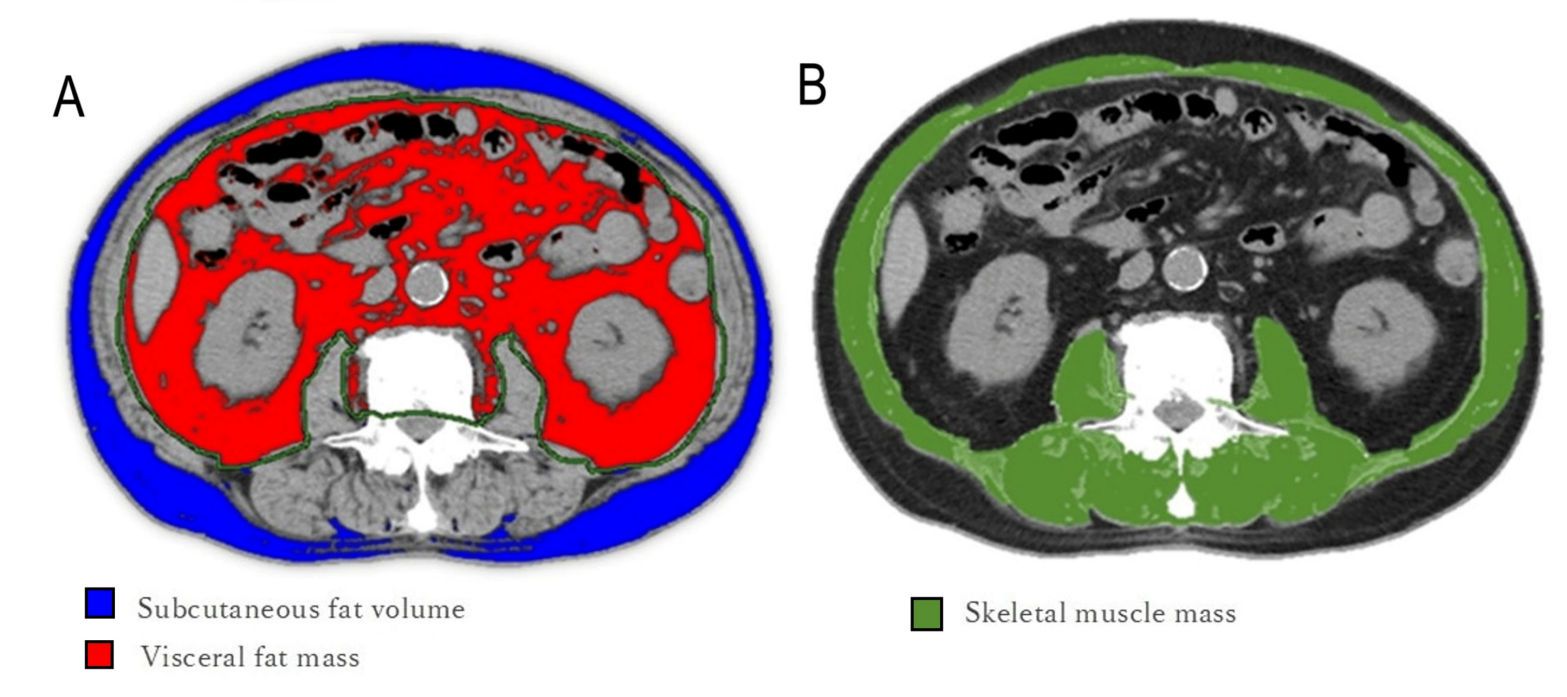
Measuring body composition using computed tomography (A) The subcutaneous and visceral fat contents were measured using L3 level images; the Hounsfield Unit (HU) value segmentation of fat was set to -150 to -70 HU. (B) The skeletal muscle mass was traced as the region of interest for all skeletal muscles at the L3 level; the cross-sectional area of the skeletal muscle at the L3 level was measured using CT value-based segmentation of -29 and 150 HU.

We investigated the relationship between fat mass, visceral fat mass, and skeletal muscle mass measured from preoperative CT images, along with preoperative blood sampling data and intraoperative body temperature. The measurements derived from CT images were specified, guided, and overseen by a radiologist. Subcutaneous and visceral fat contents were measured using Lumbar (L) 3 level images. The Hounsfield Unit (HU) value segmentation of fat was set to -150 to -70 HU. The skeletal muscle mass was traced as the region of interest (ROI) for all skeletal muscles at the L3 level. The cross-sectional area of the skeletal muscle at the L3 level was measured using CT value-based segmentation of -29 and 150 HU (Figure 2).

To standardize the estimated area, the index was calculated by dividing the heights of the two surpluses. The subcutaneous adipose tissue index (SATI) is the cross-sectional area of subcutaneous fat (cm^2^) divided by height^2^ (m^2^), and the visceral adipose tissue index (VATI) is the visceral adipose cross-sectional area (cm^2^) divided by height^2^ (m^2^). Skeletal Muscle Index (SMI) was calculated as skeletal muscle cross-sectional area (cm^2^)/height^2^ (m^2^). A Canon CT application: Fat Measurement (Canon Medical Systems Corporation, Ōtawara, Tochigi, Japan) was used for the analysis.

Statistical analysis

Statistical analysis was performed using Fisher’s exact test and the Mann-Whitney U test to compare the hypothermia and control groups. Univariate logistic regression analysis was performed to identify the factors showing significant differences. Furthermore, receiver operating characteristic (ROC) analysis was performed, and the cutoff values were calculated. Easy R (EZR) software version 1.60 (Division of Hematology, Saitama Medical Center, Jichi Medical University, Shimotsuke, Japan) was used for all statistical analyses, and the significance level was set at p < 0.05. Continuous variables were expressed as medians with ranges (minimum, maximum).

## Results

The analysis included a total of 70 patients, with 35 patients each in the hypothermia and control groups. No significant differences were observed in patient background factors between the two groups (Table 1).

**Table 1:** Comparison of patient characteristics of the two groups Categorical variables are presented as n (%) and were compared by Fisher’s exact test and continuous variables are presented as median (range) and compared by Mann-Whitney’s U test. A p-value < 0.05 is considered statistically significant. ASA-PS : American Society of Anesthesiologists Physical Status

Variable	Control Group (n = 35)	Hypothermia Group (n = 35)	P value
Sex (male), n (%)	21 (60.0%)	15 (42.85%)	0.232
Age (years), median (range)	73.00 (67.50-81.00)	74.00 (69.00-79.50)	0.972
Height (m), median (range)	159.00 (151.90-166.50)	161.70 (152.45-167.35)	0.823
Weight (kg), median (range)	62.00 (53.70, 68.15)	58.90 (49.20, 64.45)	0.222
ASA-PS (%), n (%)			0.673
Ⅰ	4 (11.4%)	2 (5.7%)	
Ⅱ	31 (88.6%)	33 (94.3%)	
Anesthesia time (minutes), median (range)	263.00 (219.50- 370.50)	265.00 (215.00-356.00)	0.916
Operation time (minutes) median (range)	209.00 (173.00-309.50)	215.00 (166.00-307.00)	0.916
Blood loss (ml), median (range)	130.00 (10.00-517.50)	40.00 (0.00-160.00)	0.066
Infusion (ml), median (range)	1650.0 (1150.0-2950.0)	1500.0 (1125.0-2200.0)	0.589

Blood data showed a significant difference in activated partial thromboplastin time (APTT) (P=0.024) (Table 2).

**Table 2:** Comparison of preoperative blood and urine test results of the two groups Mann-Whitney’s U test was used. A p-value < 0.05 is considered statistically significant. ALB: albumin; APTT: activated partial thromboplastin time; BS: blood sugar; Hb: hemoglobin; Ht: hematocrit; HbA1c: glycated hemoglobin; PT: prothrombin time; INR: international normalized ratio; RBC: red blood cell; TP: total protein; WBC: white blood count

Variable	Control group (n = 35), median (range)	Hypothermia group (n = 35), median (range)	P value
Alb (g/dl)	4.10 (3.75-4.35)	4.10 (3.65-4.40)	0.782
APTT (sec)	31.00 (29.50-33.00)	33.00 (30.50-34.00)	0.024
BS (mg/dl)	115.00 (95.00-143.00)	128.00 (102.25-152.50)	0.242
Hb (g/dl)	12.60 (10.95-13.90)	12.40 (11.25-14.10)	0.846
HbA1c (%)	5.50 (5.20-6.00)	5.80 (5.50-6.20)	0.113
Ht (%)	39.20 (32.05-41.15)	37.90 (33.25-41.65)	0.856
PT (%)	91.20 (83.60-99.20)	95.00 (83.40-102.40)	0.751
PT INR	1.06 (1.00-1.12)	1.02 (0.99-1.11)	0.541
PT (seconds)	12.10 (11.65-12.50)	11.80 (11.55-12.45)	0.584
RBC (10000.μl)	413.00 (368.00-453.00)	426.00 (370.00-469.00)	0.82
TP (g/dl)	7.20 (6.55-7.45)	7.00 (6.50-7.40)	0.592
WBC (100.μl)	61.00 (55.00-70.00)	65.00 (55.00-71.50)	0.365
Urine output (ml)	150.00 (67.50-235.00)	170.00 (117.50-285.00)	0.312

Body composition variables were compared between the hypothermia and control groups (Table 3). SATI (cm²/ⅿ²) was significantly lower in the hypothermia group (P=0.033) with a median of 27.91 cm²/ⅿ² compared to the control group with a median of 37.83 cm²/ⅿ². VATI (cm²/m²) did not significantly differ between the hypothermia (median, 32.62 cm²/ⅿ²) and control groups (median, 32.53 cm²/ⅿ²) (P=0.828). Similarly, SMI (cm²/ⅿ²) exhibited no significant differences between the hypothermia (median, 31.93 cm²/ⅿ²) and control groups (median, 34.65 cm²/ⅿ²) (P=0.19).

**Table 3:** Comparison of CT body composition index of the two groups Mann-Whitney’s U test was used. A p-value < 0.05 is considered statistically significant. SATI: Subcutaneous Adipose Tissue Index; VATI: Visceral Adipose Tissue Index; SMI: Skeletal Muscle Index

Variable	Control group (n = 35), median (range)	Hypothermia group (n = 35), median (range)	P value
SATI (cm²/ⅿ²)	37.83 (31.57-49.81)	27.91 (18.50-42.75)	0.033
VATI (cm²/ⅿ²)	32.53 (18.10-53.77)	32.62 (16.53-47.25)	0.828
SMI (cm²/ⅿ²)	34.65 (27.80-39.61)	31.93 (25.87-35.62)	0.19

**Figure 3:**
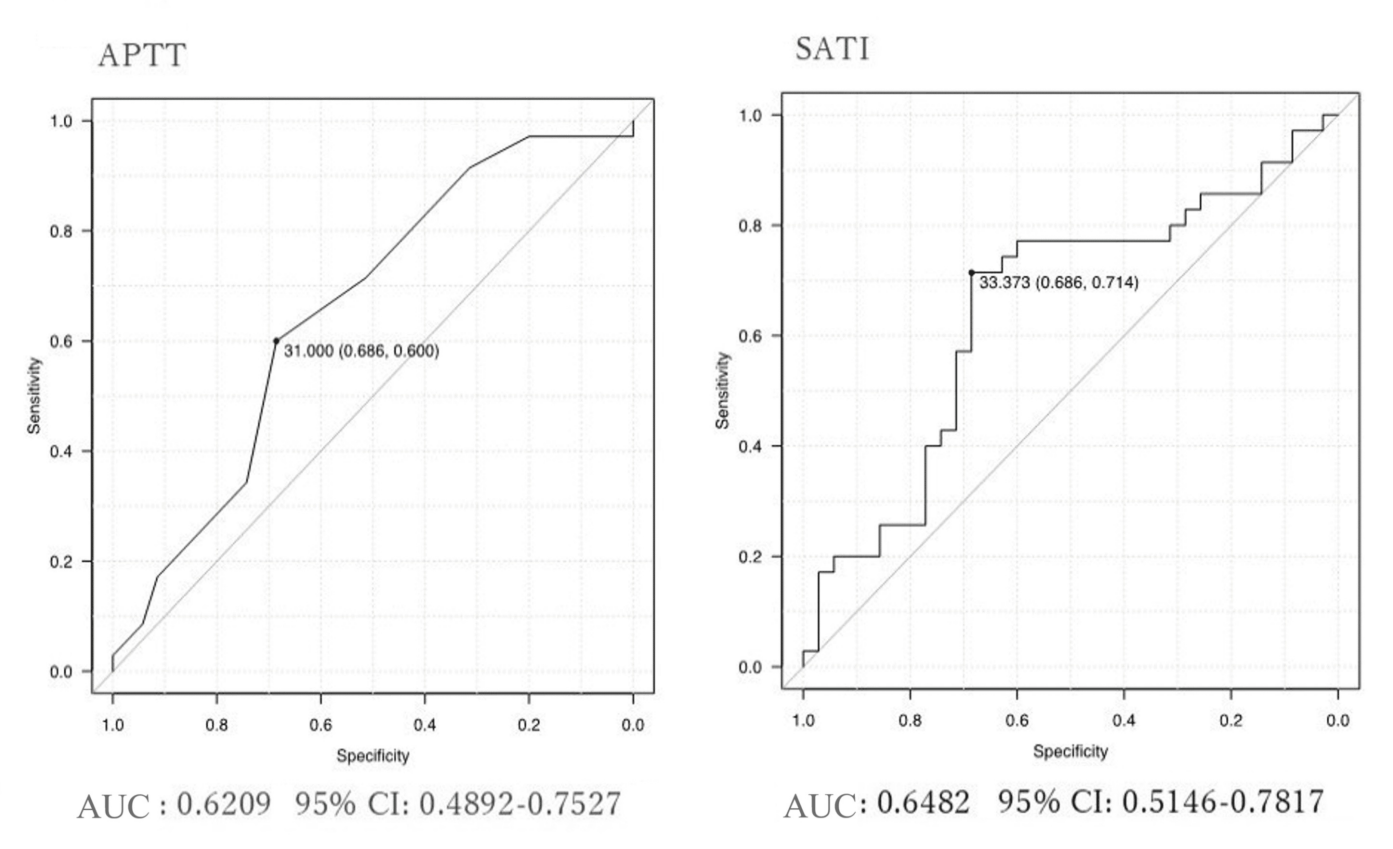
Receiver operating characteristic curves for APTT and SATI Cut-off value was determined by maximum degree of Youden index APTT: activated partial thromboplastin time; SATI: Subcutaneous Adipose Tissue Index; AUC: area under the curve

ROC analysis was performed for APTT and SATI, which revealed significant differences, to determine the optimal cutoff values. The cut-off value of APTT was chosen at 31.0 seconds, with an area under the curve (AUC) of 0.6209 (95%CI: 0.4892-0.7527), while the cut-off value of SATI was 33.373 cm²/ⅿ², with an AUC of 0.6482 (95% confidence interval (CI): 0.5146-0.7817) (Figure 3).

Univariate logistic regression analyses were performed to identify the risk factors associated with the development of hypothermia (Table 4). SATI was a significant factor in both univariate (odds ratio: 5.45, 95%CI: 1.960-15.20, P = 0.01).

**Table 4:** Logistic regression analysis of APTT and SATI A p-value < 0.05 is considered statistically significant. APTT: activated partial thromboplastin time, SATI: Subcutaneous Adipose Tissue Index

	Odds Ratio	95%CI	P Value
APTT (seconds)	0.663	0.237-1.86	0.43
SATI (cm²/ⅿ²)	5.45	1.960-15.20	0.01

## Discussion

In this study, we investigated the association between body composition, assessed using CT images, and intraoperative hypothermia during gastrointestinal surgery. The results revealed that the subcutaneous fat index was associated with intraoperative hypothermia. In contrast, visceral fat and skeletal muscle indices were not associated with intraoperative hypothermia. Previously reported risk factors for hypothermia include prolonged anesthesia and operative time, advanced age (>65 years), BMI ≥25 kg/m^2^, high ASA score, major surgery, endoscopic surgery, administration of unwarmed IV fluids, and use of epidural anesthesia [17,18].

Perioperative hypothermia is commonly defined as a body temperature <36.0℃. In a study involving gastrointestinal surgery, hypothermia developed during surgery in 46% and 41% of patients in the open and laparoscopic groups, respectively [19]. In this study, the incidence of hypothermia was 50%, which is consistent with the findings of previous reports. Additionally, even though CT is expensive and associated with radiation exposure, it is routinely performed preoperatively in gastrointestinal surgery for intra-abdominal evaluation. We believe that this quantified information is valuable for predicting intraoperative temperature decreases.

Hypothermia and SATI

In this study, we observed that SATI was a risk factor for perioperative hypothermia. Previous studies have reported that the mean body surface temperature depends on body fat composition [20]. Body temperature and subcutaneous fat levels correlated with each other. Therefore, we believe that significant differences in the subcutaneous fat index were observed in this study. The mechanism by which subcutaneous fat contributes to body temperature reduction may be related to its insulating effect, which retains body heat. However, some studies have suggested that subcutaneous fat is not associated with changes in deep body temperature during laparoscopic surgery [13]. This may be influenced by differences in surgical procedures. Although laparoscopic surgery, unlike open laparotomy, is sealed and the insulation effect of subcutaneous fat may be enhanced, it may be susceptible to pneumoperitoneum gas. Further studies are required for each surgical procedure. To date, no previous study has compared SATI with perioperative hypothermia. A previous study reported an association between body temperature control and BMI [17]. Although BMI is calculated based on height and weight, a more accurate measurement of subcutaneous fat mass may provide a more meaningful quantitative evaluation. In gastrointestinal surgery, preoperative evaluation of the abdominal region using CT is commonly performed and considered an objective and reliable method. Predicting intraoperative hypothermia using CT-derived data may benefit patients without imposing any additional burden.

Hypothermia and VATI

Visceral fat has been demonstrated to be protective against deep body temperature reduction during laparoscopic surgery [13]. In this study, no significant differences were observed in visceral fat. Although BMI values were similar between the groups, the absence of a significant difference in visceral fat may be related to factors such as differences in abdominal conditions between open and laparoscopic surgeries, the use of insufflation gas, and variation in the heating range. Continued investigation into its relationship with intraoperative hypothermia is warranted.

Hypothermia and SMI

SMI was not associated with hypothermia. Skeletal muscles play a role in heat production [21]. Primarily through shivering. However, shivering does not occur in patients under general anesthesia and therefore does not contribute to heat production. Conversely, it has been reported that reduced skeletal muscle mass lowers the body’s capacity to produce heat, leading to decreased heat production at rest [21]. If the body temperature is low before the induction of anesthesia, the risk of temperature loss due to anesthesia or surgery may be higher than that in normothermic patients. In recent years, Japan has been facing the problem of an aging society, and dealing with the elderly has become a challenge [22]. As decreased skeletal muscle mass is also a diagnostic criterion, continued investigation into its relationship with intraoperative hypothermia is warranted.

Limitations

This study has several limitations. First, it was a single-center, retrospective study, and the interpretation of the results may be influenced by confounding factors, such as variations in surgical and anesthetic procedures. Second, the sample size was small compared to that of other studies and limited to the Japanese population. Third, we did not collect detailed data regarding the surgical approach, such as whether the procedures were performed laparoscopically or via open surgery. The surgical approach may influence perioperative temperature management and patient outcomes; therefore, future studies should include this information for a more comprehensive analysis. Fourth, some patients in this study received epidural anesthesia, which is known to affect thermoregulation and may contribute to the development of perioperative hypothermia. However, due to the retrospective design and limited available data, we were unable to perform subgroup analyses based on the use of epidural anesthesia. Future prospective studies should consider evaluating the impact of epidural anesthesia on intraoperative temperature management. Furthermore, in this study, pharyngeal and bladder temperatures were used as indicators of core body temperature. However, depending on the definitions used, these measurement sites may be classified as peripheral rather than core temperatures, and there may be some variability. Although this study is novel, confounding factors such as operating room temperature, preoperative temperature, preoperative warming, intraoperative warming, measurement sites, anesthesia, and surgical techniques could not be minimized. Although the findings provide valuable insight into the relationship between body composition and intraoperative hypothermia, the limited number of participants may affect the generalizability of the results. Given the widespread use of general anesthesia across various surgical procedures and populations, larger-scale studies involving more diverse patient groups are needed to confirm and validate these findings. Future multicenter prospective studies would help enhance the external validity and broader applicability of this research.

## Conclusions

The study's primary finding is that intraoperative hypothermia is significantly associated with decreased SATI measured from preoperative CT images. This suggests SATI, due to subcutaneous fat's insulating effect, could be a valuable predictive tool for hypothermia. In contrast, no significant associations were found with VATI or SMI. Given the study's limitations (single-center, small scale), larger, multicenter prospective studies are recommended.

## References

[1] Matsukawa T, Sessler DI, Sessler AM, Schroeder M, Ozaki M, Kurz A, Cheng C (1995). Heat flow and distribution during induction of general anesthesia. Anesthesiology.

[2] Schmied H, Kurz A, Sessler DI, Kozek S, Reiter A (1996). Mild hypothermia increases blood loss and transfusion requirements during total hip arthroplasty. Lancet.

[3] Rajagopalan S, Mascha E, Na J, Sessler DI (2008). The effects of mild perioperative hypothermia on blood loss and transfusion requirement. Anesthesiology.

[4] Kurz A, Sessler DI, Lenhardt R (1996). Perioperative normothermia to reduce the incidence of surgical-wound infection and shorten hospitalization. N Engl J Med.

[5] Mahoney CB, Odom J (1999). Maintaining intraoperative normothermia: a meta-analysis of outcomes with costs. AANA J.

[6] Han SB, Gwak MS, Choi SJ, Ko JS, Kim GS, Son HJ, Shin JC (2014). Risk factors for inadvertent hypothermia during adult living-donor liver transplantation. Transplant Proc.

[7] Kasai T, Hirose M, Yaegashi K, Matsukawa T, Takamata A, Tanaka Y (2002). Preoperative risk factors of intraoperative hypothermia in major surgery under general anesthesia. Anesth Analg.

[8] Ikeda T, Ozaki M, Sessler DI, Kazama T, Ikeda K, Sato S (1999). Intraoperative phenylephrine infusion decreases the magnitude of redistribution hypothermia. Anesth Analg.

[9] Groene P, Zeuzem C, Baasner S, Hofmann-Kiefer K (2019). The influence of body mass index on temperature management during general anaesthesia-a prospective observational study. J Eval Clin Pract.

[10] Borkan GA, Gerzof SG, Robbins AH, Hults DE, Silbert CK, Silbert JE (1982). Assessment of abdominal fat content by computed tomography. Am J Clin Nutr.

[11] Cruz-Jentoft AJ, Baeyens JP, Bauer JM (2010). Sarcopenia: European consensus on definition and diagnosis: report of the European Working Group on Sarcopenia in Older People. Age Ageing.

[12] Kurz A, Sessler DI, Narzt E, Lenhardt R, Lackner F (1995). Morphometric influences on intraoperative core temperature changes. Anesth Analg.

[13] Miyazaki R, Hoka S, Yamaura K (2019). Visceral fat, but not subcutaneous fat, is associated with lower core temperature during laparoscopic surgery. PLoS One.

[14] Derstine BA, Holcombe SA, Goulson RL (2017). Quantifying sarcopenia reference values using lumbar and thoracic muscle areas in a healthy population. J Nutr Health Aging.

[15] Dindo D, Demartines N, Clavien PA (2004). Classification of surgical complications: a new proposal with evaluation in a cohort of 6336 patients and results of a survey. Ann Surg.

[16] (2016). National Institute for Health and Care Excellence: Hypothermia: prevention and management in adults having surgery. NICE Clinical Guidelines. Hypothermia: Prevention and Management in Adults Having Surgery. NICE Clinical Guidelines, No. 65.

[17] Sari S, Aksoy SM, But A (2021). The incidence of inadvertent perioperative hypothermia in patients undergoing general anesthesia and an examination of risk factors. Int J Clin Pract.

[18] Frank SM, Beattie C, Christopherson R, Norris EJ, Rock P, Parker S, Kimball AW Jr (1992). Epidural versus general anesthesia, ambient operating room temperature, and patient age as predictors of inadvertent hypothermia. Anesthesiology.

[19] Nguyen NT, Fleming NW, Singh A, Lee SJ, Goldman CD, Wolfe BM (2001). Evaluation of core temperature during laparoscopic and open gastric bypass. Obes Surg.

[20] Chudecka M, Lubkowska A, Kempińska-Podhorodecka A (2014). Body surface temperature distribution in relation to body composition in obese women. J Therm Biol.

[21] Blondin DP, Haman F (2018). Shivering and nonshivering thermogenesis in skeletal muscles. Handb Clin Neurol.

[22] Kato ET, Sato J (2023). Addressing health and demographic challenges in Japan’s ageing society. Lancet Diabetes Endocrinol.

